# The effect of Carvacrol on Enterococcus faecalis as a final irrigant

**Published:** 2009-07-06

**Authors:** Ali Nosrat, Behnam Bolhari, Mohammad Reza Sharifian, Marziyeh Aligholi, Mahsa Sadat Mortazavi

**Affiliations:** 1 *Department of Endodontics, Dental School, Rafsanjan University of Medical Sciences, Rafsanjan, Iran*; 2 *Department of Endodontics, Faculty of Dentistry/Dental Research Center, Tehran University of Medical Sciences, Tehran, Iran*; 3 *Instructor of Microbiology, School of Medicine, Tehran University of Medical Sciences, Tehran, Iran*; 4 *Student of dentistry, Dental School, Tehran University of Medical Sciences, Tehran, Iran*

**Keywords:** Carvacrol, *Enterococcus faecalis*, MTAD, Sodium hypochlorite

## Abstract

**INTRODUCTION:** Sodium hypochlorite (NaOCl) is an effective antimicrobial irrigant, however its toxic effects and deterrent odor are not ideal. Carvacrol is an edible plant extract with anti-inflammatory and anti-bacterial properties that is effective against *Enterococcus *(*E*)* faecalis*. The aim of this study was to evaluate Carvacrol's antibacterial efficacy against *E. faecalis* bacteria as a final irrigant.

**MATERIALS AND METHODS:** Forty extracted single-rooted human teeth were utilized. After mechanical preparations, samples were randomly divided into three experimental (A, B and C) and two control groups. *E. faecalis* was cultured in both experimental and positive control groups. After bacterial counting in all canals, 5.25% NaOCl, 0.6% Carvacrol emulsion and MTAD were used as final irrigants in groups A, B and C respectively. Data were analyzed using Kruskal-Wallis and Mann-Whitney *U* tests.

**RESULTS:** There was no meaningful difference in bacterial reduction between groups A and B; however, group C showed significantly lower efficacy compared to other groups (P<0.05).

**CONCLUSION:** The 0.6% Carvacrol disinfects root canals effectively. It also has anti-inflammatory qualities and therefore may be an acceptable alternative for NaOCl.

## INTRODUCTION

The main goal of endodontic treatment is disinfection of root canal system and dentinal tubules from bacteria, the main culprits of pulpal and periapical diseases (1,2).

Current preparation techniques miss areas of the root canal system, (3) and therefore, irrigation with strong antibacterial agents becomes necessary.

Sodium hypochlorite (NaOCl) is one of the most commonly used irrigants. It is a proteolytic solution which was firstly used by Dakin with a concentration of 0.5% (4).

Lower concentrations of NaOCl (0.5-1%) can eliminate intra-canal bacteria and necrotic tissues; also concentrations of over 1% may not alter efficacy (5,6).

NaOCl has presented some disadvantages such as tissue toxicity, unfavorable taste and odour. This solution is neither capable of thorough elimination of smear layer nor complete disinfection of root canal system (7,8); additionally, it can cause irritation if it passes through the apical foramen (9,10).

Recently, a new irrigant called MTAD has been introduced and used in endodontic treatments. MTAD is the combination of tetracycline isomer (Doxycycline), citric acid and a detergent (11). An *in vitro* study has shown that MTAD is more effective than NaOCl in eliminating *Enterococcus (E) faecalis* (11).

However, MTAD is more expensive than NaOCl and also can cause reddish discoloration of tooth due to doxycycline oxidation (12).

Carvacrol (thymol isomer) is one of the ingredients of origanum oil and essential oil which are edible plant oils used in food products. Carvacrol is a liquid with spicy odor and has a colorless to yellow hue. This plant extract is a Food and Drug Administration (FDA) approved food additive (FDA reg. no 172.5151).

Carvacrol is insoluble in water but soluble in alcohol and ether. It has a broad spectrum of antibacterial activity; it works by inhibiting ATPase activity and increasing the non-selective permeability of bacterial cell membranes. Therefore, it not only inhibits microbial colonization but also makes microbes more sensitive to antibacterial agents (13,14).

Carvacrol has antibacterial effect against *Enterobacteriaceae* family including *Escherichia Coli*, *Salmonella Enteritidis* and *Salmonella Essen* (15).

Antibacterial effect of Carvacrol and its isomer thymol against six ATCC standard bacterial strains including *E. faecalis* has been proved (16).

Carvacrol also has anti-inflammatory effects. It can restrain neutrophilic elastase enzyme and suppress prostaglandin production (17,18).

From our pilot study we concluded that minimum bactericidal concentration (MBC) of Carvacrol against *E. faecalis* is 0.6%.

The bactericidal effect of Carvacrol irrigant on *E. faecalis* in root canal system has not been studied yet.

The aim of this *in vitro* study was to evaluate 0.6% Carvacrol emulsion efficiency, as a final irrigant on *E. faecalis* bacteria in comparison with 5.25% NaOCl and MTAD.

## MATERIALS AND METHODS

Forty single-canalled maxillary incisors and mandibular premolars were selected and stored in normal saline in order to prevent dehydration. All samples were decoronated at CEJ level using high speed diamond fissure bur (Diatech, Heerbrugg, Switzerland). Working lengths were determined with K-file size #10 or 15 (Dentsply Maillefer, Tulsa, Ok) and were in the range between 12-15-mm. Root canal instru-mentation was performed as outlined below: After preserving patency of canal with K-file size #15 (Dentsply Maillefer, Tulsa, Ok), Gates Glidden Drills sizes 1-3 (Dentsply Maillefer, Tulsa, Ok) were used passively to prepare the coronal third of root canals. Then preparation of apical third was carried out by using passive step back method with hand K-files up to size #30. Next, Profile rotary system (Dentsply, Tulsa, Ok) sizes 20-0.06, 25-0.06, 30-0.06 were used to reach the working length of the canals. Irrigation was performed between each instrument using 2 mL of normal saline.

In order to eliminate smear layer, 10cc of 17% EDTA (Aria Dent, Asia Chem., Teb Co., Tehran, Iran) was used for one minute and then the canals were irrigated with 5 cc of 5.25% sodium hypochlorite (Golrang factory, Pakshoo Co., Iran). Roots were mounted in transparent acrylic resin and autoclaved to achieve sterilization.

Five roots were randomly selected and used as the negative control group and transferred into the incubator. *E. faecalis* (ATCC 29212) was cultured in aerobic condition on 5% defibrinated sheep blood/Brain Heart Infusion (BHI) agar medium and then suspended in BHI broth. The cell suspension was adjusted spectrophotometrically according to 0.5 McFarland standard (19). During the 4-weeks period, microbial suspension was replenished in the canals every 3 days except for negative control group which received sterilized normal saline every 3 days.

After this period, five roots were randomly selected among samples as positive controls. Other roots were divided into three groups. We used sterilized paper cones size 20 (Sendoline Solna, Sweden) to verify the presence of bacteria inside the canals, then scheduled procedures were carried out for each group.

In group A, 5cc of 5.25% NaOCl was used for root canals irrigation and maintained within the canals for 5min. Subsequently, the canals were irrigated with 5cc of sterile normal saline in order to eliminate NaOCl, and sampled with paper cones size 20. Canals were not dried before sampling.

In group B, samples were irrigated with 0.6% Carvacrol emulsion and left for 5 minutes. After 5 minutes, the canals were irrigated with 5cc sterile normal saline in order to eliminate Carvacrol and then sampled with paper cones size 20. Canals were not dried before sampling.

**Table 1:** The number of Enterococcus faecalis bacterial colonies before and after 5 minutes of application of irrigants inside canals.

Samples	Group A		Group B		Group C	
	Before	After	Before	After	Before	After
1	38×10^5^	1200	1500	0	86×10^4^	2000
2	30×10^4^	0	123×10^4^	2000	84×10^4^	10400
3	78×10^5^	0	37×10^5^	0	38×10^4^	2000
4	54×10^4^	0	12×10^5^	800	17×10^4^	0
5	11×10^4^	0	48×10^5^	0	46×10^4^	1600
6	-	-	55×10^5^	1140	43×10^4^	600
7	22×10^5^	0	11×10^5^	60	33×10^4^	600
8	65×10^5^	0	25×10^5^	0	11×10^4^	2700
9	31×10^4^	0	81×0^5^	40	36×10^5^	13600
10	27×10^3^	0	56×10^3^	0	33×10^4^	1000

In group‏ C, MTAD was prepared according to the manufacturer's instructions; the canals were irrigated with 1cc MTAD, and left for 5 minutes. Then irrigation continued with remaining 4cc MTAD. Immediately after, the canals were irrigated with 5cc sterile normal saline in order to eliminate MTAD and sampling carried out as same as other two experimental groups.

In the positive control group, canals were irrigated with 5cc sterile normal saline in order to emulate the procedure in experimental groups. Sampling was carried out identically to the experimental groups.

**Table 2:** The number of Enterococcus faecalis bacterial colonies in control groups before and after irrigation with sterile normal saline

Samples	Positive control		Negative control	
	Before	After	Before	After
1	41×10^5^	28×10^5^	0	0
2	45×10^5^	55×10^4^	0	0
3	23×10^5^	19×10^5^	0	0
4	25×10^4^	15×10^4^	0	0
5	35×10^5^	20×10^5^	0	0

Samples with negative results were separated for dentinal shavings. This was carried out by cutting the acrylic base of the teeth with a sterilized disc and spatula into two halves; one half of the tooth was separated from the acrylic base and the apical portion left intact in the acryl. Since there was direct access to the roots, it was possible to cut the dentinal wall completely from the coronal third to apical area without contamination using a sterilized round bur (Diatech, Heerbrugg, Switzerland). Therefore, dentinal shavings could be carried in special plates and cultured in tubes containing BHI agar. After 48-hours incubation, tubes were evaluated for microorganism growth by turbidity test and positive cases recorded.

The same procedure was carried out in the negative control group for accuracy.

Co-variance analysis test was used after final irrigation of canals in various groups to determine the number of bacterial colony in comparison with the previous co-variate count. Percentage of bacterial growth reduction in each sample was calculated in each group and an average was measured. Kruskal-wallis test was used to compare bacterial reduction percentage in each group and Mann-Whitney *U* test with bon ferroni correction was used for pair comparisons.

## RESULTS

One sample in group A was omitted due to external contamination. The results of sampling before and after canal irrigation are presented in Tables 1 and 2.

Comparing groups A, B and C with the positive control group showed that the amount of bacterial reduction in each group was higher than control positive group indicating a significant difference (P<0.05). Comparison of groups A and B in terms of bacterial reduction showed that they had no significant difference (P>0.05). The amount of bacterial reduction in groups A and B is higher than group C indicating a significant difference (P<0.05). Bacterial reduction percentages are shown in Table 3.

Group A had 8 roots, group B had 5 roots and group C had 1 root with absence of bacteria after irrigation (dentinal walls were used to prepare dentine shavings for culture).

The results of these cultures compared to that of the control groups are shown in Table 4.

## DISCUSSION

In a pilot study, MBC of Carvacrol against *E. faecalis* (ATCC 29212) was determined to be 0.6%. *E. faecalis* was chosen for this study as this bacterium has proved to be the most common organism in root canals with failed endodontic treatment (20,21).

Haapasolo *et* *al.* (22) showed that *E.* *faecalis* required 4-weeks culturing in order to contaminate dentinal tubules.

**Table 3:** Bacterial reduction percentages are shown individually in each group

***Group***		***n***	***Minimum***	***Maximum***	***Mean***	***SD***
A	Before	9	27×10^3^	78×10^5^	2043×10^3^	2982882.16
	After	9	0	12×10^2^	133.33	4×10^2^
	Percent reduced	9	99.68	100	99.96	0.105
	Valid N(listwise)	9				
B	Before	9	56×10^3^	55×10^5^	2320666.7	1922452.6
	After	9	0	2×10^3^	448.89	718.61
	Percent reduced	9	99.83	100	99.97	0.05
	Valid N(listwise)	9				
C	Before	9	11×10^4^	36×10^5^	1016666.7	1738231.86
	After	9	0	136×10^2^	3611.11	4890.41
	Percent reduced	9	97.54	100	99.43	0.79
	Valid N(listwise)	9				
Control	Before	9	25×10^4^	45×10^5^	2522×10^3^	1938406.56
	After	9	15×10^4^	28×10^5^	1576×10^3^	1214960.95
	Percent reduced	9	34.88	40	38.05	2.16
	Valid N(listwise)	9				

**Table 4:** The result of cultured dentinal shavings which are prepared from canal walls with absence of bacteria

**Dentinal Shaving**	**Contaminated with Bacteria**	**Absence of Bacteria**
Group A	2	6
Group B	3	2
Group C	0	1
Positive control	5	0
Negative control	0	5

Therefore, we cultured *E. faecalis* for 4 weeks inside the canals. The presence of bacteria within dentinal shavings of root canal walls in some of the experimental samples after the final irrigation, indicated that effective smear layer removal and subsequent colonization and entrance of bacteria into dentinal tubules, had been carried out successfully.

In the present study, after biomechanical preparation, the smear layer was eliminated by 17% EDTA and by irrigating with 5.25% NaOCl; the *E. faecalis* was then cultured. This procedure was carried out for all groups. Therefore bacteria and irrigants had the same opportunity to enter the dentinal tubules.

Negative results in negative controls indicated the accuracy of initial sterilization. The 38% decrease in *E. faecalis* counts in the positive control group can be attributable to washing out of microorganisms during saline irrigation, and before the second sampling. Note that sterile normal saline has no antibacterial effect. The present study demonstrated that *E. faecalis* can be eliminated effectively (99%) from the canals following 5-minutes irrigation with 0.6% Carvacrol. Gill *et al.* (13) and Helander *et al.* (14) studied the ATP changes at the cellular level in bacteria induced by Carvacrol. They observed that these bacteria released ATP and concluded that Carvacrol destroyed cell membranes and inhibited ATPase activity.

NaOCl is an irrigants with widely used in endodontic treatment (5,6). Interestingly, this study showed no meaningful differences between 0.6% Carvacrol emulsion and 5.25% NaOCl in 5-min application. We have illustrated that 5.25% NaOCl and 0.6% Carvacrol emulsion can effectively eradicate intra-canal bacteria with 5-min applications compared to Biopure MTAD. An *In vitro* study showed that final irrigation with Biopure MTAD in comparison with 5.25% NaOCl is more effective in obliterating bacteria inside the root canals (11). However the methods employed were different with our study, producing different results. Dunavant *et al.* (23) observed meaningful difference between NaOCl (1% and 6%) and Biopure MTAD; NaOCl is more effective against *E. fcaecalis* biofilm elimination, agred with the present study. Johal *et al.* (24) showed that root canal irrigation with 5.25% NaOCl and 15% EDTA is more effective than 1.3% NaOCl and Biopure MTAD.

## CONCLUSION

The disadvantages of NaOCl have induced much search for an ideal replacement. The anti-inflammatory and antibacterial properties of Carvacrol emulsion make it a promising ideal irrigant. Further studies are needed to overcome some problems *e.g.* discovering a water soluble variant which can sustain the antibacterial and anti-inflammatory features. Also, the effect of irrigating with Carvacrol emulsion on the sealing ability of root filling materials is unclear and should be analyzed.
